# A comparison of the effectiveness of cyclophosphamide, leflunomide, corticosteroids, or conservative management alone in patients with IgA nephropathy: a retrospective observational study

**DOI:** 10.1038/s41598-018-31727-5

**Published:** 2018-09-12

**Authors:** Shasha Chen, Qing Yin, Song Ren, Xiang Zhong, Wei Wang, Guisen Li, Li Wang

**Affiliations:** Renal Division and Institute of Nephrology, Sichuan Academy of Medical Sciences and Sichuan Provincial People’s Hospital, Medical School of University of Electronic Science and Technology of China, Chengdu, 610072 China

## Abstract

To compare the long-term efficacy of corticosteroids (P) alone or in combination with cyclophosphamide (CTX), leflunomide (LEF), or Angiotensin-convertase inhibitors or angiotensin II receptor blockers (ACEI/ARB) in treatment for IgA nephropathy (IgAN), 311 patients with IgAN were identified. Therapeutic effectiveness (including progression, partial remission, complete remission) and combined renal endpoint (defined as 30% reduction in eGFR or ESRD) were compared based on different therapies. After immunosuppressive and ACEI/ARB treatment, the levels of eGFR, proteinuria and albumin were significantly improved at the last follow-up, the extent of improvement of eGFR, proteinuria, and albumin was more notable in P + CTX group and P + LEF group. 41%, 52.2%, 55.3% and 55.2% in P + CTX, P + LEF, P and ACEI/ARB group achieved complete remission, respectively. Multivariate regression analysis indicated that only proteinuria (Relative risk (RR) 0.82(0.72–0.94), P = 0.004) and tubular atrophy/interstitial fibrosis (RR 0.26(0.13–0.57), P = 0.001) were predictors for complete remission. The optimal cutoffs of eGFR was 47.085 ml/min/1.73 m^2^ predicting renal function recovery in P + CTX therapy. In conclusion, tubular atrophy/interstitial fibrosis and massive proteinuria were poor predictors for complete remission in IgAN, it appears as though patients may have benefited from immunosuppressive treatment but that comparison to a well-matched contemporary control group or, ideally, a randomized controlled clinical trial, would be required to show this.

## Introduction

Immunoglobin A nephropathy (IgAN) is the most frequent primary glomerulonephritis worldwide, especially in China. It accounts for approximately 40–50% of primary glomerulonephritis^1,2^. IgAN has various clinical and pathological manifestations and corresponding considerable variations in prognosis. It is a relatively benign disease, however, the long term prognosis should not be considered mild, because, after 20 years of disease progression, 20–50% of the patients reach end stage renal disease^3–6^. A number of immunosuppressive agents have been used for treatment of IgAN, including calcineurin inhibitors^7,8^, azathioprine^9^, mycophenolate mofetil^10,11^, cyclophosphamide^11,12^, leflunomide^13,14^, corticosteroids^12,15–17^, etc. Many studies have only compared two treatment regimens for a relatively small sample size. However, the protective role of immunosuppressive therapy was still in controversy^18^. It is essential to find out patients who may benefit from immunosuppressive treatment and the proper choice of immunosuppressive therapy or supportive care^3^. We therefore retrospectively analyzed 311 patients with IgAN treated with CTX, LEF, P or conservative management alone in our center to identify long-term therapeutic effectiveness and renal outcome, and explore the prognostic factors for renal function recovery in patients with reduced renal function.

## Materials and Methods

### Patients

All patients with biopsy-proven IgAN in Sichuan Provincial People’s Hospital from 2006 to 2014 were reviewed. The baseline and follow-up data of the patients were obtained from the database of Glomerulonephritis Registry in our department. The diagnosis of IgAN was based on diffuse IgA-dominant depositions in the glomerular mesangial region and mesangial electron dense deposition in electron microscopy. The exclusion criteria included secondary IgAN (such as lupus nephritis, Henoch-Schönlein purpura, chronic liver cirrhosis and other autoimmune disorders), crescentic IgAN (defined as ≥50% crescentic glomeruli on kidney biopsy), or minimal change disease with glomerular IgA deposits, with acute kidney injury, aged <18 years at biopsy or with follow-up <6 months. The protocol for this study was approved by medical ethics committee of the Sichuan Academy of Medical Sciences and Sichuan Provincial People’s Hospital, and informed written consent for the treatment they received was obtained from all of the identified patients. All treatment studies were performed in accordance with relevant guidelines and regulations.

### Clinical and laboratory data

Baseline clinical data included gender, age, disease duration, blood pressure (measured at the morning of the biopsy day), gross hematuria at the time of biopsy. Laboratory data included 24-h urinary protein excretion (Upr), serum albumin (Alb), serum creatinine (Scr), uric acid (UA), liver enzymes, and estimated glomerular filtration rate (eGFR) calculated using the equation of CKD-EPI(CKD Epidemiology Collaboration) formula^19^. Scr and Upr during follow-up are also collected.

### Renal pathology

The scoring sheet was based on the Oxford classification of IgAN^20^, and seven pathological variables, namely mesangial hypercellularity (M), endocapillary proliferation (E), segmental sclerosis or adhesion (S), tubular atrophy/interstitial fibrosis (T), crescents (C), glomerulus necrosis (N), and vascular changes(hyaline degeneration and intimal thickening) were assessed. Tubulointerstitial lesions, including tubular atrophy, interstitial inflammation, and interstitial fibrosis, were semi-quantitatively sorted into the following categories: none (score;0), mild (1; <25%), moderate (2; 25–49%), and severe (3; ≥50%)^21^.

### Treatment

Most patients in the immunosuppressant groups also received ACEI/ARB therapy. Patients with persistent proteinuria (more than 1 g/day) and a eGFR of more than 50 ml/min per 1.73 m^2^ received steroid therapy with or without ACEI/ARB. Those with moderate(1–3 g/day) to severe(>3 g/day) proteinuria, sever acute tubulointerstitial(3; ≥50%) or acute glomerular injury(crescents, glomerular necrosis) received immunosuppressive agents including LEF or CTX. P was given at a dosage of 0.8–1.0 mg/kg/day (The maximum dosage of 60 mg per day) for 8 weeks, then gradually tapered to stop within 6–9 months. Intravenous CTX was given twice a month for 6 months and the dosage was 0.5–0.75 g/m^2^ body surface area per month according to a modified National Institutes of Health (NIH) protocol^22^. LEF was orally administered with 50 mg/day for 3 days, reduced to 20 mg/day for 3–6 months, and subsequently tapered^23,24^. All the patients who were treated with CTX or LEF also received prednisone followed the previous protocol. ACEI/ARB were used in a dosage titration method until they reached the maximum tolerable dosage. Major adverse events included elevated liver enzymes, leucopenia, respiratory infection, and steroid diabetes were retrospective reviewed of outpatient and inpatient electronic medical record system.

### Definition

ESRD was defined as eGFR <15 mL/min/1.73 m^2^, initiation of dialysis for more than 3 months or transplantation)^25^. Therapeutic effectiveness included progression, partial remission and complete remission based on proteinuria and renal function^26^, assessed after six months of treatment. Progression was defined as proteinuria ≥1 g/day and declining eGFR, partial remission was defined as proteinuria ≤1 g/day and stabilization of eGFR, complete remission was defined as a reduction of proteinuria to <0.3 g/day and renal function recovery. Normal range Scr was defined as Scr <104 umol/L. Renal function recovery was defined as improvement of eGFR by at least 20% or back to normal range.

### Statistical analysis

All data were analyzed by using statistical software SPSS 19.0. Normally distributed variables were expressed as mean ± SD. Non-parametric variables were expressed as median and interquartile range. Categorical variables were expressed in percentages. All parameters were compared by Chi-square test, Fisher test for categorical data and the Student’s t-test, one-way analysis of variance (ANOVA) analysis or Kruskal-Wallis test for continuous data. Survival rate was estimated with the Kaplan–Meier method. The outcome event was defined as a combined renal endpoint: 30% reduction in eGFR or ESRD. Univariate analysis followed by multivariate logistic regression analysis was used to evaluate the cumulative probability of complete remission in the whole group and renal function recovery in patients with reduced renal function in subgroups. All baseline variables were entered in the initial model and maintained if *P* < 0.10. ROCs of the above significant risk factors by logistic regression were drawn to determine the optimal cutoffs predicting recovery of renal function. The function to calculate the probability of this event was as follows:

$${\bf{P}}={\bf{b0}}+{\bf{b1}}\times {\bf{X}}+{\bf{b2}}\times {{\bf{X}}}^{{\bf{2}}}+{\bf{b3}}\times {{\bf{X}}}^{{\bf{3}}}$$

where P is the probability of improved renal function, and x is the baseline eGFR that was identified as an independent risk factor for improved renal function on multivariate logistic regression analysis.

## Results

### Baseline clinical and laboratory features of IgAN patients treated with different therapy

A total of 311 patients with biopsy-proven IgAN diagnosed from January 2006 to January 2014 in Sichuan Provincial People’s Hospital were identified in the retrospective study. 192 (61.7%) were females and 119 (38.3%) were males, with an average age of 36.4 ± 11.0 years old. P + CTX group had a much higher level of Scr (*P* < 0.001) and a lower level of eGFR (*P* < 0.001) than other groups. LEF group, steroid group and especially P + CTX group had more severe hypoproteinemia (*P* < 0.001) and heavier proteinuria than ACEI/ARB group. ACEI/ARB group had a significantly lower percentage of nephrotic proteinuria than other three groups (Table 1).

**Table 1:** Demographic, clinical, and laboratory characteristics at renal biopsy.

	P + CTX (n = 85)	P + LEF (n = 73)	P (n = 80)	ACEI/ARB (n = 73)	*P* value
Age - yr.	37.2 ± 9.7	36.3 ± 12.2	36.3 ± 11.6	35.9 ± 10.7	0.897
Female sex - no. (%)	54(63.5%)	45(61.6%)	50(62.5%)	43(58.9%)	0.944
Duration of disease -mo.	4(3–7)	4(3–6)	4(2–6)	3(2–4)	0.002
MAP (mmHg)	95.4 ± 12.5	95.5 ± 13.7	98.9 ± 15.5	97.4 ± 14.9	0.361
Hypertension (%)	29(34.1%)	21(28.8%)	22(27.5%)	15(20.5%)	0.305
Gross hematuria (%)	24(28.6%)	16(21.9%)	19(22.5%)	22(30.1%)	0.555
eGFR(ml/min/1.73 m^2^)^abce^	64.7 ± 35.3	81.1 ± 30.4	89.3 ± 30.4	91.6 ± 29.2	p < 0.001
Cr (umol/L)^abc^	123.1 ± 49.3	99.0 ± 43.1	99.9 ± 43.6	86.8 ± 33.8	p < 0.001
UA (mmol/L)	384.8 ± 113.7	355.0 ± 116.8	372.3 ± 109.2	361.8 ± 105.0	0.456
Proteinuria (g/d)^cef^	1.5(0.9–2.5)	1.8(0.7–2.9)	1.2(0.9–2.6)	0.8(0.4–1.3)	p < 0.001
nephrotic range proteinuria%^cef^	16.5	17.8	17.5	1.4	0.007
Hb(g/dL)	125.9 ± 22.1	129.0 ± 21.5	132.8 ± 20.8	132.0 ± 20.5	0.157
Alb (g/L)^cef^	36.8 ± 6.9	37.7 ± 5.8	38.2 ± 7.8	42.9 ± 5.2	p < 0.001

^a^*P* < 0.05 between CTX and LEF group, ^b^*P* < 0.05 between CTX and P group, ^c^*P* < 0.05 between CTX and ACEI/ARB group,

^d^*P* < 0.05 between LEF and P group, ^e^*P* < 0.05 between LEF and ACEI/ARB group,

^f^*P* < 0.05 between P and ACEI/ARB group

MAP, mean arterial pressure; eGFR, estimated glomerular filtration rate; Cr, creatinine; UA, uric acid; Hb, hemoglobin; Alb, albumin.

### Pathological features

The scoring sheet was based on the Oxford classification of IgAN, P + CTX group had the highest mesangial hypercellularity score (M) (*P* < 0.001), highest proportion of global and segmental glomerulosclerosis (*P* < 0.001), most severe tubular atrophy/interstitial fibrosis lesions (*P* < 0.001), most severe interstitial lymphocyte and monocyte infiltration and most prominent vascular lesions (*P* < 0.001) than other three groups. While the ACEI/ARB group had the least prominent glomerular, tubular, interstitial and vascular lesions than other three groups (*P* < 0.001) (Table 2).

**Table 2:** Pathological characteristics at renal biopsy.

	P + CTX (n = 85)	P + LEF (n = 73)	P (n = 80)	ACEI/ARB (n = 73)	*P* value
Mesangial hypercellularity score					0.001
≤0.5	33.3%	39.7%	55.0%	63.0%	
>0.5	66.7%	60.3%	45.0%	37.0%	
Endocapillary hypercellularity					0.111
absent	51.2%	56.2%	66.3%	67.1%	
present	48.8%	43.8%	33.8%	32.9%	
Segmental glomerulosclerosis					0.100
absent	46.4%	60.3%	65.0%	56.2%	
present	53.6%	39.7%	35.0%	43.8%	
Tubular atrophy/interstitial fibrosis					p < 0.001
none (0)	9.7%	38.6%	52.2%	45.5%	
mild (1)	71.0%	47.4%	39.1%	47.0%	
moderate (2)	17.7%	12.3%	7.2%	7.6%	
severe (3)	1.6%	1.8%	1.4%	0%	
Interstitial inflammation					0.028
0	8.1%	29.8%	27.5%	31.8%	
0–20%	72.6%	56.1%	63.8%	60.6%	
20–50%	17.7%	12.3%	7.2%	7.6%	
>50%	1.6%	1.8%	1.4%	0%	
Global glomerulosclerosis (%)^c^	22.6 ± 13.7	16.4 ± 9.9	14.8 ± 7.0	11.5 ± 6.7	0.010
Segmental sclerosis (%)^abc^	11.5 ± 8.0	5.6 ± 4.5	7.0 ± 4.2	5.3 ± 4.1	0.009
Crescents (%)	7.8 ± 5.3	9.2 ± 4.6	5.5 ± 5.2	5.7 ± 5.9	0.159
Capillary necrosis (%)	5.9%	1.4%	0%	1.4%	0.051
Vasculopathy^abc^	82.3%	49.1%	44.9%	30.3%	p < 0.001

^a^*P* < 0.05 between CTX and LEF group, ^b^*P* < 0.05 between CTX and P group,

^c^*P* < 0.05 between CTX and ACEI/ARB group, ^d^*P* < 0.05 between LEF and P group,

^e^*P* < 0.05 between LEF and ACEI/ARB group, ^f^*P* < 0.05 between P and ACEI/ARB group.

### Outcome and survival from combined renal endpoint in patients with different therapy

92.9%, 91.8% and 92.5% of patients in P + CTX, P + LEF and P group received ACEI/ARB, during a median follow-up of 31.9 months (range 18.1–47.9), 5 patients (1.6%) of the 311 cases entered ESRD and 22 patients (7.1%) reached the combined endpoint. 17 patients (5.5%) had ≥40% reduction in eGFR and 13 patients (4.2%) had ≥50% reduction in eGFR. (*P* < 0.001) The ACEI/ARB group had the highest level of eGFR than other groups. P + CTX group had higher annual increase rate and value of eGFR, higher proportion of 30% and 50% rise in eGFR than ACEI/ARB group (Table 3).

**Table 3:** Follow-up and outcome data.

	P + CTX (n = 85)	P + LEF (n = 73)	P (n = 80)	ACEI/ARB (n = 73)	*P* value
Duration of follow-up — mon	38.2(22.3–59.8)	38.2(30.8–57.0)	28.6(15.8–36.4)	20.5(12.5–35.0)	p < 0.001
RAS blockers %	92.9	91.8	92.5	100	0.006
MAP(mmHg)	93.3 ± 9.8	91.1 ± 9.3	90.1 ± 10.9	90.7 ± 10.7	0.392
eGFR (ml/min per 1.73 m^2^)^abc^	76.4 ± 34.1	90.2 ± 30.5	94.7 ± 28.8	92.9 ± 29.1	0.001
UA(mmol/L)	436.0 ± 340.5	373.8 ± 119.6	390.5 ± 111.5	382.3 ± 98.1	0.507
Cr (umol/L)^abc^	115.6 ± 95.3	93.4 ± 74.7	82.3 ± 40.2	86.1 ± 40.5	0.009
Proteinuria (g/d)	0.4(0.2–1.2)	0.4(0.2–0.9)	0.3(0.1–0.7)	0.3(0.2–0.6)	0.248
Alb (g/L)	47.9 ± 4.0	43.7 ± 4.5	42.7 ± 4.9	43.1 ± 5.9	0.532
Annual change in eGFR	4.7	1.8	3.5	−1.1	0.128
ESRD%	3.5%	1.4%	0%	1.4%	0.248
≥50% rise in eGFR,%^abc^	28.2%	11.0%	7.5%	8.2%	p < 0.001
≥30% rise in eGFR,%^abc^	44.7%	26.0%	18.8%	16.4%	p < 0.001
Mean initial prednisone dose(g)	52.8 ± 5.3	42.7 ± 7.4	54.3 ± 6.2	/	

annual change in eGFR: having positive value if there was an increase and negative value if there was a decline in eGFR.

^a^*P* < 0.05 between CTX and LEF group, ^b^*P* < 0.05 between CTX and P group,

^c^*P* < 0.05 between CTX and ACEI/ARB group, ^d^*P* < 0.05 between LEF and P group,

^e^*P* < 0.05 between LEF and ACEI/ARB group, ^f^*P* < 0.05 between P and ACEI/ARB group.

**Figure 1:**
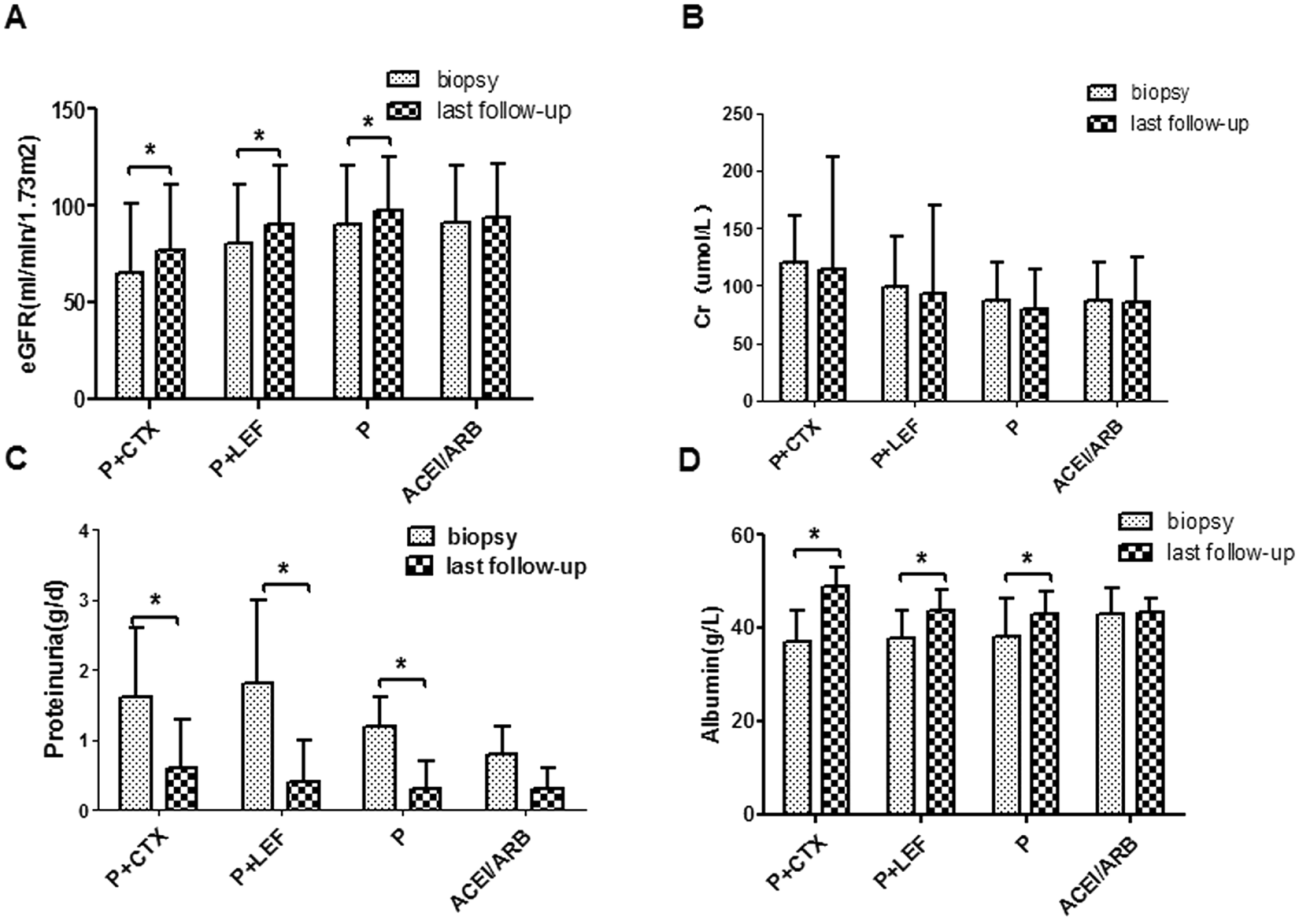
Comparison of clinical parameters (**A**). eGFR (**B**). Scr (**C**). proteinuria (**D**). Albumin at renal-biopsy and those at the last time of follow-up.  data at renal-biopsy;  data at the last time of follow-up. *Biopsy time vs last follow-up, p < 0.05.

**Figure 2:**
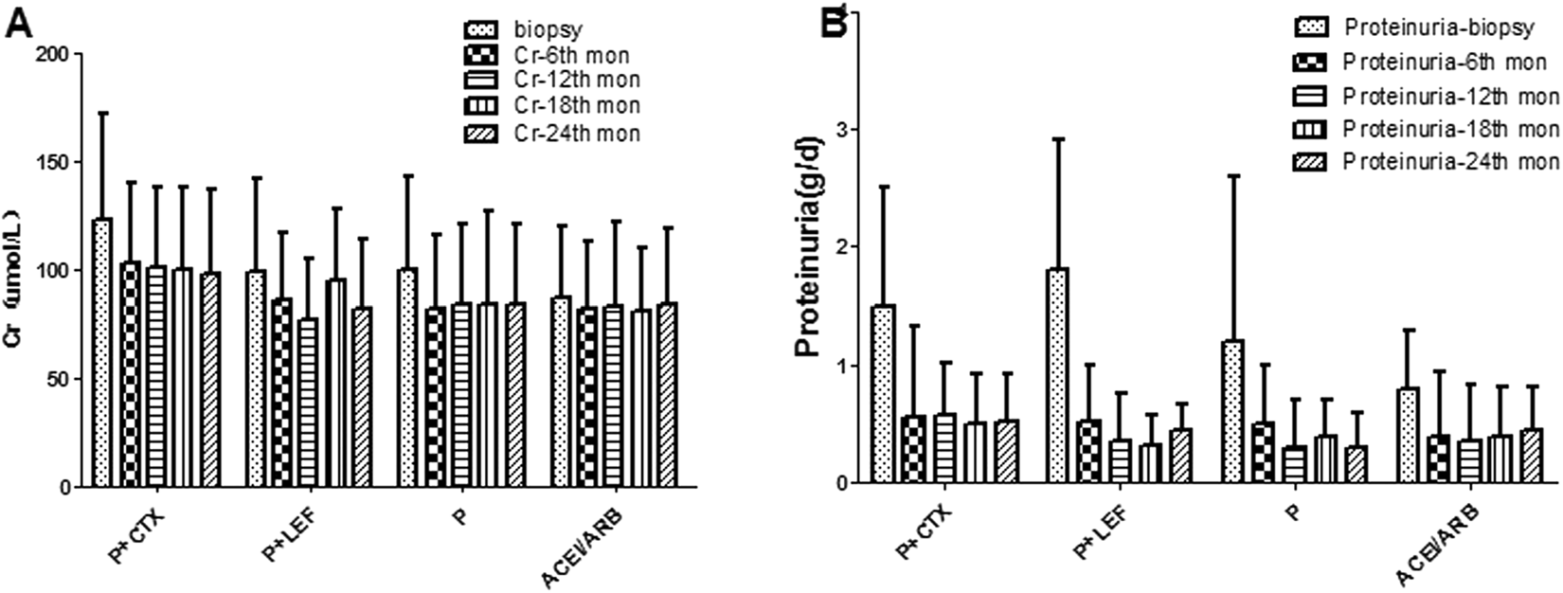
Comparison of clinical parameters (**A**). Scr and (**B**). proteinuria at renal-biopsy and those at 6^th^, 12^th^, 18^th^, 24^th^ months of follow-up.

**Figure 3:**
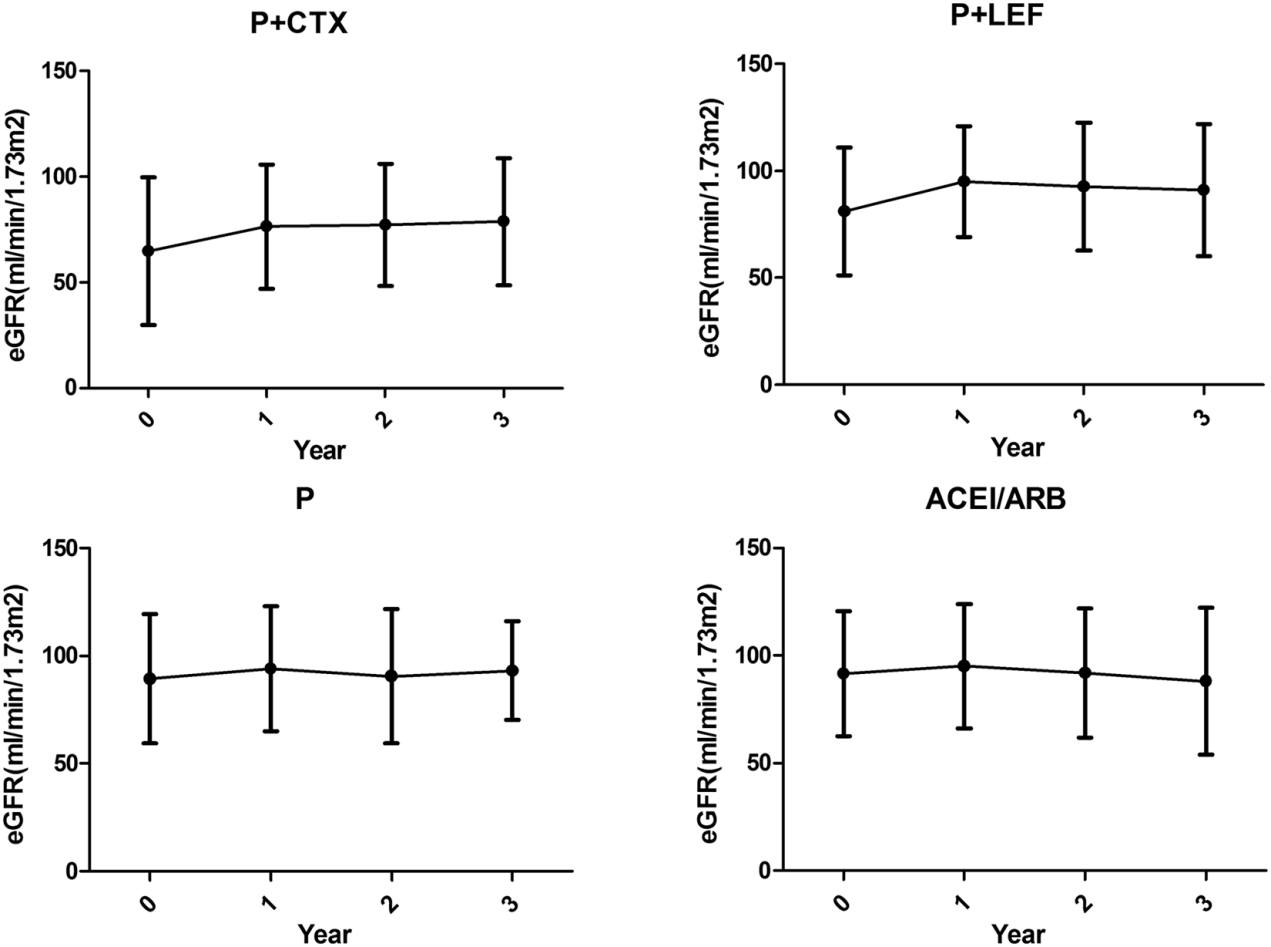
Comparison of eGFR at renal-biopsy and those at 1st, 2nd, 3^rd^ year of follow-up.

Figure 1 compared clinical parameters of renal-biopsy and those at the last follow-up. The ACEI/ARB group had the lowest level of proteinuria than other groups. After immunosuppressive and ACEI/ARB treatment, the levels of eGFR, proteinuria and albumin were significantly improved at the last time of follow-up than those at renal biopsy, and the extent of improvement of eGFR, proteinuria, and albumin was more notable in P + CTX group and P + LEF group than others. Proteinuria decreased significantly from 1.6 g/d to 0.6 g/d after P + CTX therapy, and from 1.8 g/d to 0.4 g/d after P + LEF therapy. Then we identified those followed for at least 2 years (200 patients including 46,50,58 and 46 in P + CTX, P + LEF, P and ACEI/ARB group) to compare the changes of Scr, proteinuria and eGFR before and after treatment. Figure 2 compared Scr concentration and proteinuria at renal-biopsy and 6^th^, 12^th^, 18^th^, 24^th^ months of follow-up, P + CTX group, P + LEF group and steroid treatment effectively improved proteinuria than ACEI/ARB group. Figure 3 showed that annual change of eGFR was 4.7, 1.8, 1.8 and −1.1 ml/min/1.73 m^2^/year.

**Figure 4:**
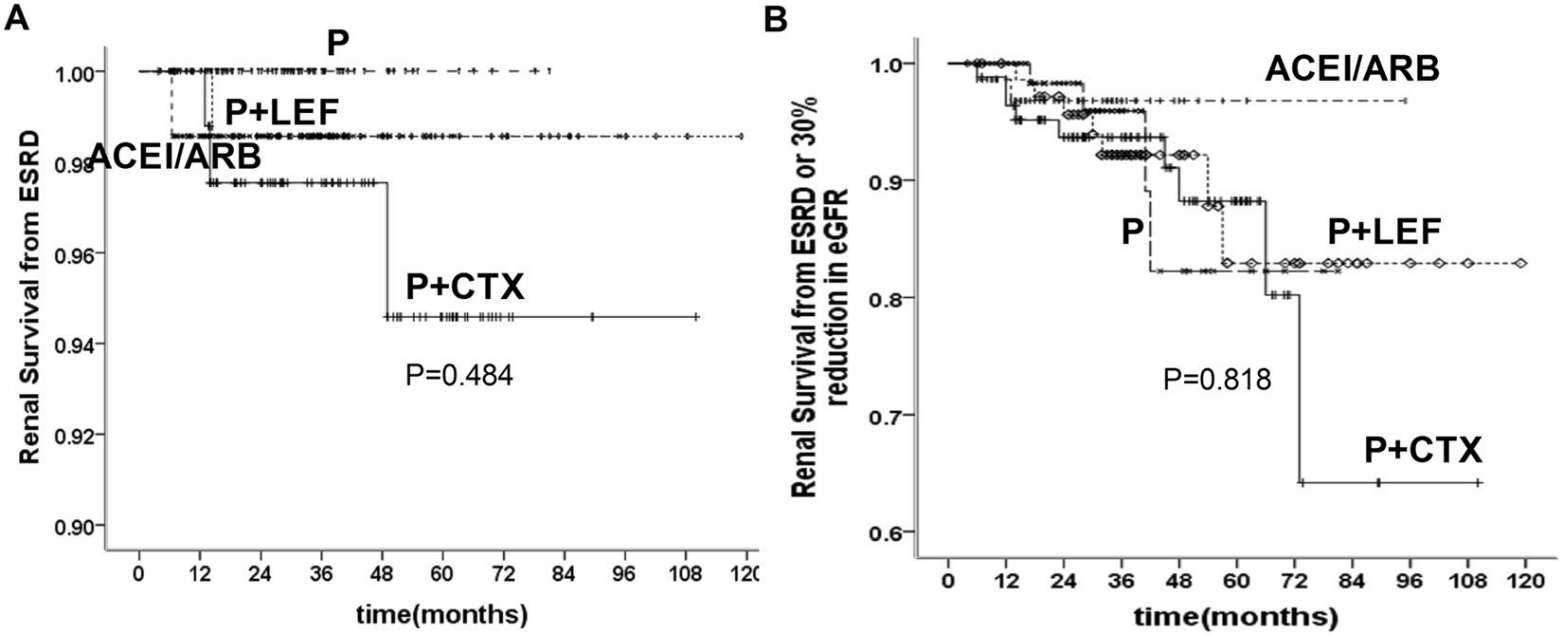
Comparison of renal survival from ESRD (**A**) and ESRD or 30% reduction in eGFR (**B**) in patients with different therapy.

Regarding combined renal endpoint, P + CTX group had the worst outcome than other groups and steroid group had the best outcome than other groups. The 3-, 5- and 10-year cumulative renal survival rates from the combined renal endpoint, calculated by Kaplan–Meier method, were 96.0%, 93.1% and 66.2% in P + CTX group, 94.7%, 88.8% and 88.8% in P + LEF group, 98.3%, 98.3% and 98.3% in steroid group, 96.7%, 96.7% and 96.7% in ACEI/ARB group respectively (Fig. 4).

### Predictors for complete remission in IgAN with different therapies

41%, 52.2%, 55.3% and 55.2% in P + CTX, P + LEF, steroid and ACEI/ARB group achieved complete remission, respectively (Table 4). Univariate logistic regression indicated that hypertension (Relative risk (RR) 82.012, P = 0.006), eGFR (RR 1.011, P = 0.004), proteinuria (RR 0.776, P = 0.001), UA (RR 0.997, P = 0.015), M (RR 1.877, P = 0.007), S (RR 0.590, P = 0.023), T lesions (RR 0.356, P = 0.004) and immunosuppressants therapy (RR 1.707, P = 0.020) were predictors for complete remission in the whole group. Multivariate regression analysis indicated that only proteinuria (RR 0.824, P = 0.004) and tubular atrophy/interstitial fibrosis (RR 0.260, P = 0.001) were predictors for complete remission (Table 5).

**Table 4:** Efficiency of Glucocorticoids, Cyclophosphamide, Leflunomide and RAS blockers in treatment for IgA nephropathy.

	P + CTX (n = 83)	P + LEF (n = 69)	P (n = 76)	ACEI/ARB (n = 67)
Progression n, %	21(25.3%)	15(21.7%)	12(15.8%)	18(26.9%)
Partial remission n, %	28(33.7%)	18(26.1%)	22(28.9%)	12(17.9%)
Complete remission n, %	34(41%)	36(52.2%)	42(55.3%)	37(55.2%)

**Table 5:** Predictors for complete remission in IgAN with different therapies by univariate and multivariate logistic regression.

	Univariate		Multivariate	
	RR (95% CI)	P-value	RR (95% CI)	*P* value
Age	1.007(0.987–1.028)	0.502	—	—
Sex	1.348(0.851–2.134)	0.203	—	—
Hypertension	2.012(1.218–3.324)	0.006	—	—
Gross hematuria	1.061(0.636–1.771)	0.820		
UA((mmol/L))	0.997(0.995–0.999)	0.015		
Albumin(g/L)	1.050(1.015–1.086)	0.005		
Proteinuria (g/d)	0.776(0.673–0.895)	0.001	0.821(0.717–0.939)	0.004
Creatinine(mg/dl)	0.990 (0.985–0.996)	0.001	—	—
eGFR (ml/min/1.73 m^2^)	1.011 (1.003–1.018)	0.004	—	—
M	1.877(1.191–2.958)	0.007	—	—
E	0.831(0.527–1.313)	0.428	—	—
S	0.590(0.374–0.929)	0.023	—	—
T	0.356(0.210–0.602)	0.004	0.26(0.133–0.507)	0.001
Crescents (%)	0.983(0.960–1.007)	0.165	—	—
Vasculopathy	0.669(0.406–1.104)	0.116	—	—

RR = relative risk; 95% CI = 95% confidence interval; All data obtained at time of renal biopsy.

At renal biopsy, 128 cases (41.2%) had impaired renal function, after immunosuppressants and ACEI/ARB treatment, 75 patients (58.6%) achieved renal function recovery, and 57 patients’ (44.5%) Scr concentration returned to normal range. We afterwards respectively explored the risk factors for renal recovery in patients with reduced renal function in each group.

After a complete or partial remission, four patients in P + CTX group had a relapse of nephrotic proteinuria and achieved a complete or partial remission after P + LEF therapy (P was given at a dosage of 0.8–1.0 mg/kg/day, LEF was orally administered with 20 mg/day),five patients in P + LEF relapsed, two of them were treated with prednisone at a dose of 0.8–1.0 mg/kg/day, and three were treated with P + LEF(with the initial dose), two patients in P group relapsed and were re-induced with 0.8–1.0 mg/kg/day dose of prednisone.

### Predictors for renal function recovery in P + CTX group

The cumulative dosage of CTX in P + CTX group was 7.5 ± 0.3 g. 53 cases in P + CTX group had impaired renal function, in which 21 patients’ (39.6%) Scr concentration returned to normal range and 31 cases (58.5%) achieved renal function recovery. At the time of biopsy, univariate logistic regression indicated that gross hematuria (RR 8.478, P = 0.049), eGFR (RR 1.070, P = 0.020) and proteinuria (RR 0.588, P = 0.020) were predictors for renal function recovery in CTX group. Multivariate regression analysis indicated that only eGFR (RR 1.094, P = 0.027) was independently associated with renal function recovery (Table S1).

**Figure 5:**
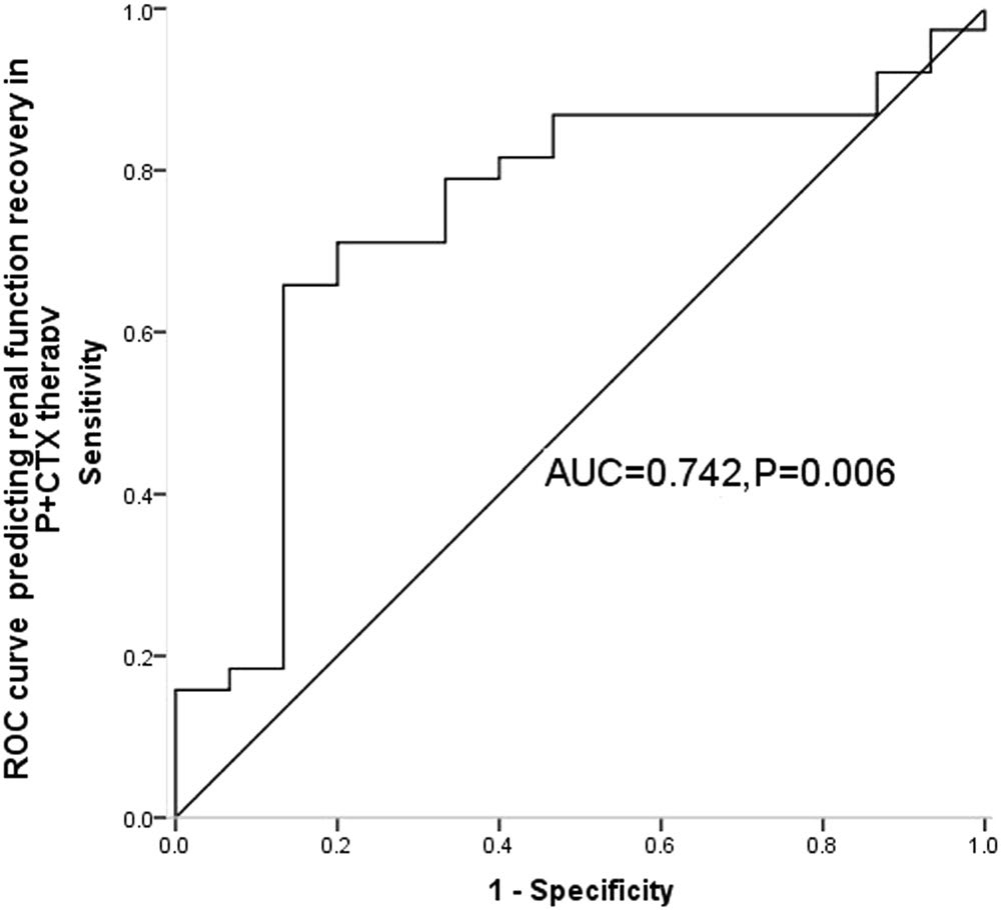
The receiver operating characteristic (ROC) curve for the GFR cutoff of renal function recovery in P + CTX therapy.

ROC of eGFR was drawn to determine the optimal cutoffs predicting renal function recovery. The area under the ROC (AUC) was 0.742 (p = 0.005, 95% CI 0.590–0.894), indicating that the initial eGFR at renal biopsy had high predictive accuracy for renal function recovery for IgAN patients with P + CTX treatment. The optimal cutoffs of eGFR was 47.085 ml/min/1.73 m^2^ (specificity = 65.8%, sensitivity = 86.7%) (Fig. 5).

### Model for renal function recovery in P + CTX group

We used the strongest predictive factor, the initial eGFR as the variable, and demonstrated that the association between the probability of renal function recovery and eGFR fitted a cubic shape, we calculated the model for the prediction of renal function recovery as follows:

$${\bf{P}}={\bf{0.051}}+{\bf{0.012}}\times {\bf{eGFR}}+{\bf{1}}{\bf{.75E}}-{\bf{4}}\times {\bf{eGF}}{{\bf{R}}}^{{\bf{2}}}+(-{\bf{2}}{\bf{.26E}}-{\bf{6}})\times {\bf{eGF}}{{\bf{R}}}^{{\bf{3}}}$$

(R^2^ = 0.999, *P* = < 0.001) (eGFR, ml/min/1.73 m^2^, calculated by CKD-EPI equation^19^).

**Figure 6:**
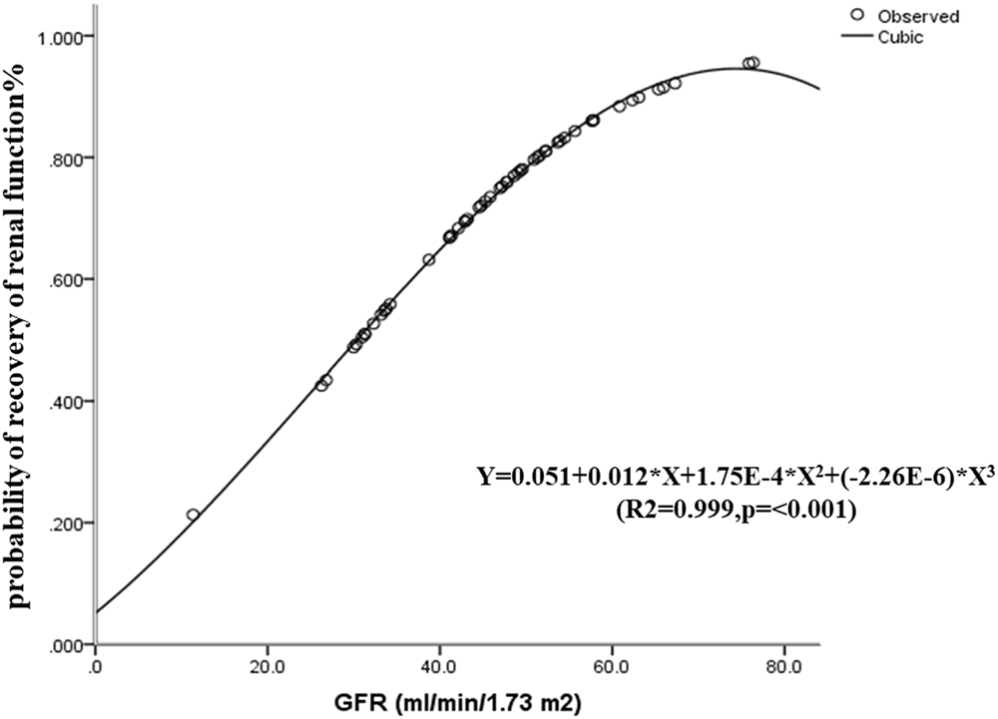
The logistic regression curve of the association between the probability of renal function recovery and eGFR in P + CTX group. A value of 0 means that the patient did not achieved renal function recovery, whereas 1.0 means that 100% of the patient achieved renal function recovery. The curve is cubic-shaped. In patients with eGFR >51.2 ml/min/1.73 m^2^, the cumulative probability of renal function recovery is >80%.

When the initial eGFR was higher than 51.2 ml/min/1.73 m^2^, the cumulative probability of renal function recovery was above 80% (Fig. 6). In the cohort (n = 53), 19 patients had an initial eGFR higher than 51.2 ml/min/1.73 m^2^, and 16 patients (84.2%) achieved renal function recovery during follow-up.

### Predictors for renal function recovery in P + LEF group

31 cases in P + LEF group had impaired renal function, in which 15 patients’ (48.4%) Scr concentration returned to normal range and 18 cases (58.1%) achieved renal function recovery. At the time of biopsy, univariate logistic regression indicated that gross hematuria (RR 0.069, P = 0.022), and Scr concentration (RR 0.962, P = 0.039) were predictors for renal function recovery in P + LEF group. Multivariate regression analysis indicated that only gross hematuria (RR 0.045, P = 0.016) was an independent predictor of poor response to P + LEF therapy (Table S2).

### Predictors for renal function recovery in P group

23 cases in P group had impaired renal function, in which 12 patients’ (52.2%) Scr concentration returned to normal range and 15 cases (65.2%) achieved renal function recovery. At the time of biopsy, univariate and multivariate logistic regression indicated that crescents (RR 0.677, P = 0.049) predicted poor response to corticosteroid therapy Table S3).

### Major adverse events

Elevated liver enzymes, leucopenia and respiratory infection occurred in 4 cases (4.7%), 4 cases (4.7%) and 2 cases (2.4%) in P + CTX group. Elevated liver enzymes, alopecia and respiratory infection occurred in 2 cases (2.7%), 2 cases (2.7%) and 1 case (1.4%) in P + LEF group. Elevated liver enzymes and steroid diabetes occurred in 3 cases (3.8%), 3 cases (3.8%) in P group. No significant difference was found of the incidence of adverse effects among the three groups. None of the patients in either group developed incident diabetes, hyperkalemia or serious infections that would cause drug withdrawal or termination. After symptomatic treatment, those with liver function injury, leucopenia and infection recovered and continued the therapy.

## Discussion

IgA nephropathy has remained the most common form of primary glomerulonephritis leading to chronic kidney disease in developed countries. ESRD occurs in 30% to 40% of patients 20 to 30 years after the first clinical presentation. Identification of high-risk patients and who may benefit from immunosuppressive treatment is important^27–30^. The therapeutic measures range from no therapy with the only need of regular follow-up, to supportive therapy eventually associated with low dose immunosuppression, to immunosuppressive treatment in the attempt to avoid the evolution to end stage renal disease^31^. There is an ongoing debate over the efficacy and safety of immunosuppressive agents other than ACEI/ARB monotherapy in IgAN. However, the current evidence about the different therapies remains to be elucidated^31^. Here we included four most widely applied therapies in our center to explore the treatment response, predictors for complete remission and adverse effects of immunosuppressive agents in IgAN.

ACEI/ARB were given in more than 90% but not all of patients in three suppressants groups. Though ACEI/ARB group had higher incidence of complete remission, those with cytoxic therapy had more severe renal injury and pathological features before treatment.

The efficiency of CTX with corticosteroids has been principally examined in small retrospective studies concerning patients with progressive renal deterioration or with crescentic IgAN^32–36^. Due to insufficient evidence and uncertain risk-benefit balance, a possible role is suggested by the guidelines only for patients with crescentic IgAN and rapidly decreasing renal function, analogous to the treatment of anti-neutrophil cytoplasmic antibody (ANCA) vasculitis^32,34^.

Our study found that patients who received CTX therapy presented with more proteinuria, microscopic hematuria and hypoalbuminemia and severe pathologic lesions than those with P + LEF, P and ACEI/ARB, however, P + CTX was effective in reducing urinary protein levels and conserve kidney function in patients with IgAN, although we excluded crescentic IgAN. 44.7% and 28.2% had more than 30% and 50% rise in eGFR. 58.5% of 53 cases in P + CTX group with impaired renal function achieved renal recovery during follow-up.

Multiple regression in our study revealed that eGFR was an independent risk factor for renal recovery in IgAN patients with P + CTX treatment. We further used eGFR as variable to draw ROC and demonstrated that the optimal cutoff for predicting renal recovery was eGFR 47.1 ml/min/1.73 m^2^, supporting that P + CTX should be considered promising therapeutic agents for the treatment of IgAN with reduced renal function. Using logistic curves, we developed a simple model based on eGFR to predict renal recovery of IgAN treated with P + CTX. In this model, a plot of the association between the probability of renal recovery and the initial eGFR formed a cubic-shaped curve, the probability of recovery in kidney function goes down if the GFR is less than 70 ml/min, and when the initial eGFR was higher than 51.2 ml/min/1.73 m^2^, the cumulative probability of renal function recovery was above 80%., indicating that cyclophosphamide is useful if eGFR is more than 47 ml/min, CTX therapy may not achieve a promising effect when eGFR is less than 47 ml/min, this finding needs large, prospective studies to confirm.

Leflunomide^14^ is an immunosuppressive agent inhibiting T- and B-cell functions which has long been used in rheumatology. Its mechanism of action involves inhibition of dihydroorotate dehydrogenase^37,38^, as well as a number of tyrosine kinase signaling molecules involved with immune function. Leflunomide appears to be a promising treatment for lupus nephritis^39^, but there are limited data supporting the use of leflunomide as an investigational therapy for progressive IgA^40^. Our study demonstrated that LEF can effectively reduce proteinuria, as compared with the steroid therapy, similar to previous studies^13,14,41^. In addition, P + LEF group in our cohort had higher annual increase rate and value of eGFR, higher proportion of 30% and 50% rise in eGFR than P group, with almost identical baseline clinicopathological features. Gross hematuria was identified to be an independent predictor of poor response to P + LEF therapy. Our results revealed that more than half of patients with reduced renal function in P + LEF group achieved renal recovery after treatment. Therefore, P + LEF may be considered promising therapeutic agents for the treatment of IgAN^41^, especially in those without gross hematuria, but this conclusion is limited by lack of a control group of untreated patients, and should be investigated further in large sample size, high-quality studies.

Several studies have been performed to prove the effectiveness of corticosteroid therapy in IgAN^42–46^, while other studies did not confirm a steroid related beneficial effects^47^ or highlight the problem of corticosteroid side effects^48^. STOP-IgA^12^ study showed that the addition of immunosuppressive therapy to intensive supportive care did not significantly improve the outcome and may increase adverse effects. Our study suggested that adding corticosteroid and immunosuppressants to RAS blockers resulted in a better reduction of proteinuria, a higher rate of renal function recovery and a higher incidence of side effects in comparison with administering RAS blockers alone, even P group in our cohort had heavier proteinuria and severe hypoproteinemia than ACEI/ARB group.

There were some limitations to this study. Firstly, this was a single-center retrospective observational analysis. It was difficult to control for all factors that may affect renal survivorship. Secondly, treatment failure and previous renal biopsy data, which could possibly influence the outcomes, was not included because many records were lost or incomplete. Thirdly, we did not add different treatments into our multivariate regression analysis because the treatment assignment was not blinded and unified, which was based on the patients’ financial conditions and different physicians’ clinical experiences. Fourthly, ACEI/ARB and P group had a shorter period of follow-up compared to the P + LEF and P + CTX group, this could be attributed to less severe baseline clinicopathological manifestations and better prognosis than other groups, resulting in greater loss to follow-up. In addition, as a retrospective study, we didn’t include a control group of untreated patients to compare with treated groups, thus it is not known what would have happened for these patients who have not been treatment. Any improvements in renal function or proteinuria may have been due to ACE/ARB treatment, BP control, spontaneous improvement or other factors and is not necessary due to the immunosuppressive treatment. The retrospective design also limited our study with regard to adverse events. We could only include major adverse events that were mentioned in the outpatient and inpatient hospital electronic records.

In conclusion, tubular atrophy/interstitial fibrosis on renal biopsy and massive proteinuria were poor predictors for complete remission in IgAN. Reduced eGFR, gross hematuria and crescents were predictors for a lower rate of renal function recovery with CTX, LEF and corticosteroid therapy, respectively. It appears as though patients may have benefited from immunosuppressive treatment but that comparison to a well-matched contemporary control group or, ideally, a randomized controlled clinical trial, would be required to show this.

## Electronic supplementary material

supplementary material
