# Supplementary material for: A comparison of the effectiveness of cyclophosphamide, leflunomide, corticosteroids, or conservative management alone in patients with IgA nephropathy: a retrospective observational study
Source: Sci Rep. 2018 Sep 12;8:13662. doi: 10.1038/s41598-018-31727-5 (PMC6135814; doi:10.1038/s41598-018-31727-5)
Supplement: Supplementary file 1 — supplementary material [file 41598_2018_31727_MOESM1_ESM.doc]

**A comparison of the** **effectiveness of cyclophosphamide, leflunomide, corticosteroids, or conservative management alone in patients with IgA nephropathy: a retrospective observational study**

Shasha Chen, Qing Yin, Song Ren, Xiang Zhong, Wei Wang, Guisen Li*, Li Wang

Renal Division and Institute of Nephrology, Sichuan Academy of Medical Sciences and Sichuan Provincial People’s Hospital, Medical School of University of Electronic Science and Technology of China (Chengdu, 610072, China)

Corresponding Author：Guisen Li

Email address: guisenli @163.com

**Table S1. Predictors for renal function recovery in P+CTX group by univariate and multivariate logistic regression**

|  | **Univariate** |  | **Multivariate** |  |
| --- | --- | --- | --- | --- |
|  | **RR (95% CI)** | **P-value** | **RR (95% CI)** | ***P* value** |
| Age | 0.938(0.877-1.003) | 0.063 | - | - |
| Sex | 0.824(0.244-2.775) | 0.754 | - | - |
| Hypertension | 0.51(0.152-1.711) | 0.276 | - | - |
| Gross hematuria | 8.478(1.002-71.732) | 0.049 |  |  |
| Proteinuria (g/d) | 0.588(0.376-0.919) | 0.020 | - | - |
| Creatinine(mg/dl) | 0.992 (0.979-1.006) | 0.268 | - | - |
| eGFR (ml/min/1.73 m^2^) | 1.07(1.011-1.132) | 0.020 | 1.094(1.01-1.185) | 0.027 |
| M | 1.444(0.418-4.987) | 0.561 | - | - |
| E | 2.062(0.611-6.967) | 0.244 | - | - |
| S | 1.571(0.473-5.225) | 0.461 | - | - |
| T | 1.234(0.442-3.445) | 0.688 | - | - |
| Crescents (%) | 1.134(0.969-1.327) | 0.118 | - | - |
| Vasculopathy | 7.0(0.952-51.445) | 0.056 | - | - |

RR= relative risk; 95% CI= 95% confidence interval; All data obtained at time of renal biopsy.

**Table S2. Predictors for renal function recovery in P+LEF group by univariate and multivariate logistic regression**

|  | **Univariate** |  | **Multivariate** |  |
| --- | --- | --- | --- | --- |
|  | **RR (95% CI)** | **P-value** | **RR (95% CI)** | **P-value** |
| Age | 1.032(0.968-1.101) | 0.335 | - | - |
| Sex | 3.333(0.682-16.295) | 0.137 | - | - |
| Hypertension | 1.125(0.243-5.207) | 0.880 | - | - |
| Gross hematuria | 0.069(0.007-0.680) | 0.022 | 0.045(0.004-0.567) | 0.016 |
| Proteinuria (g/d) | 1.087(0.743-1.589) | 0.667 | - | - |
| Creatinine(mg/dl) | 0.962 (0.926-0.998) | 0.039 | - | - |
| eGFR (ml/min/1.73 m^2^) | 1.039(0.978-1.103) | 0.214 | - | - |
| UA(mmol/L) | 0.991(0.981-1.002) | 0.099 | - | - |
| M | 1.556(0.308-7.854) | 0.593 | - | - |
| E | 0.857(0.205-3.579) | 0.833 | - | - |
| S | 0.742(0.175-3.148) | 0.686 | - | - |
| T | 0.487(0.143-1.656) | 0.249 | - | - |
| Crescents (%) | 0.992(0.931-1.057) | 0.806 | - | - |
| Vasculopathy | 0.525(0.091-3.034) | 0.472 | - | - |

RR= relative risk;; 95% CI= 95% confidence interval; All data obtained at time of renal biopsy.

**Table S3. Predictors for renal function recovery in P group by univariate and multivariate logistic** **regression**

|  | **Univariate** |  | **Multivariate** |  |
| --- | --- | --- | --- | --- |
|  | **RR (95% CI)** | **P-value** | **RR (95% CI)** | **P-value** |
| Age | 1.042(0.953-1.140) | 0.367 | - | - |
| Sex | 1.111(0.190-6.492) | 0.907 | - | - |
| Hypertension | 1.111(0.190-6.492) | 0.907 | - | - |
| Gross hematuria | 1.750(0.151-20.23) | 0.654 | - | - |
| Proteinuria (g/d) | 0.947(0.721-1.244) | 0.696 | - | - |
| Creatinine(mg/dl) | 0.989 (0.962-1.016) | 0.424 | - | - |
| eGFR (ml/min/1.73 m^2^) | 1.009(0.949-1.073) | 0.766 | - | - |
| M | 0.667(0.118-3.755) | 0.646 | - | - |
| E | 2.00(0.298-13.435) | 0.476 | - | - |
| S | 0.606(0.097-3.788) | 0.592 | - | - |
| T | 0.704(0.153-3.250) | 0.653 | - | - |
| Crescents (%) | 0.677(0.459-0.999) | 0.049 | 0.677(0.459-0.999) | 0.049 |

RR= relative risk;; 95% CI= 95% confidence interval; All data obtained at time of renal biopsy.
